# Neuronal Network Activation Induced by Forniceal Deep Brain Stimulation in Mice

**DOI:** 10.3390/genes16020210

**Published:** 2025-02-09

**Authors:** Bin Tang, Zhenyu Wu, Qi Wang, Jianrong Tang

**Affiliations:** 1 Jan and Dan Duncan Neurological Research Institute, Texas Children’s Hospital, Houston, TX 77030, USA; bin.tang@bcm.edu (B.T.); zwu@bcm.edu (Z.W.); qi.wang@bcm.edu (Q.W.); 2 Department of Pediatrics, Baylor College of Medicine, Houston, TX 77030, USA

**Keywords:** fornix, limbic system, deep brain stimulation, learning and memory, *c*-Fos, neural circuit

## Abstract

**Background:** The fimbria-fornix is a nerve fiber bundle that connects various structures of the limbic system in the brain and plays a key role in cognition. It has become a major target of deep brain stimulation (DBS) to treat memory impairment in both dementia patients and animal models of neurological diseases. Previously, we have reported the beneficial memory effects of chronic forniceal DBS in mouse models of intellectual disability disorders. In Rett syndrome and CDKL5 deficiency disorder models, DBS strengthens hippocampal synaptic plasticity, reduces dentate inhibitory transmission or increases adult hippocampal neurogenesis that aids memory. However, the underlying neuronal circuitry mechanisms remain unknown. This study we explored the neural network circuits involved in forniceal DBS treatment. **Methods:** We used acute forniceal DBS-induced expression of *c*-Fos, an activity-dependent neuronal marker, to map the brain structures functionally connected to the fornix. We also evaluated the mouse behavior of locomotion, anxiety, and fear memory after acute forniceal DBS treatment. **Results:** Acute forniceal DBS induces robust activation of multiple structures in the limbic system. DBS-induced neuronal activation extends beyond hippocampal formation and includes brain structures not directly innervated by the fornix. **Conclusions:** Acute forniceal DBS activates multiple limbic structures associated with emotion and memory. The neural circuits revealed here help elucidate the neural network effect and pave the way for further research on the mechanism by which forniceal DBS induces benefits on cognitive impairments.

## 1. Introduction

Deep brain stimulation (DBS) may block or modify the faulty signals and provide substantial benefits for patients with various neurologic and neuropsychiatric diseases [1]. Selecting brain targets for DBS depends on the main symptoms to be treated. The fornix is a C-shape, white-matter tract bundle in the limbic system. It is the major efferent and afferent tract that connects the hippocampus to various brain structures and plays an important role in cognition and episodic memory [2]. Fornix has become a potential candidate target for DBS to treat memory impairment in dementia patients [3,4,5,6]. In mouse models of intellectual disability disorders, we reported the beneficial memory effects of chronic forniceal DBS in Rett syndrome and *Cdkl5* mutant mice [7,8]. Others have also reported that patterned forniceal stimulation improves spatial memory performance in an experimental rat model of dementia [9]. The mechanisms underpinning forniceal DBS-induced memory benefits; however, they remain unclear, and are likely multifold; for instance, through the modulation of gene expression [10], synaptic transmission and plasticity [7,8], hippocampal inhibitory activity [8,11], and hippocampal local field potential oscillations [9]. At the neural network level, DBS not only alters the neural activity of the targeted brain structure but also modulates related neural network activity. Two major and nonexclusive hypotheses have been proposed as to how DBS confers to its benefits: (1) it silences stimulated neurons and (2) it introduces a new activity pattern in the neural network. The first theory stems from the observation that, functionally, DBS produces the same effect as a lesion would do in the stimulated area. For example, DBS evokes powerful membrane depolarization that interferes with the ability of individual neurons to respond to inputs, resulting in an informational lesion [12]. The second hypothesis proposes that DBS drives an activity into the circuit that may propagate and consequently modify the pathological spontaneous activity in other nuclei [13]. Yet, the neural circuit related to forniceal DBS remains widely unknown. DBS at the fornix, a fiber tract with widely reciprocal projections in the limbic system, may enhance or attenuate the activity in multiple limbic structures and even other downstream brain regions. Therefore, screening the neural circuit is critical to elucidating its circuit mechanism of action. For this goal, we mapped the expression of activity-regulated immediate early gene *c*-Fos [14,15] in the whole brain of wild-type mice following acute forniceal DBS or sham treatment. We also evaluated the effect of acute forniceal DBS on locomotion, anxiety levels, learning and memory, and pain threshold.

## 2. Materials and Methods

### 2.1. Animals

Adult female wild-type mice (FVB.129 background) were maintained on a 12 h light–12 h dark cycle (light on at 7:00 a.m.) with standard mouse chow and water ad libitum in our on-site American Association for Laboratory Animal Science-accredited facility. Up to 5 mice per cage were group-housed before surgery and individually housed with nesting material in the cage after surgery in a room maintained at 22 °C. All the experimental procedures and tests were conducted during the light cycle. All research and animal care procedures were approved by the Baylor College of Medicine IACUC.

### 2.2. Surgery and DBS

**Figure 1:**
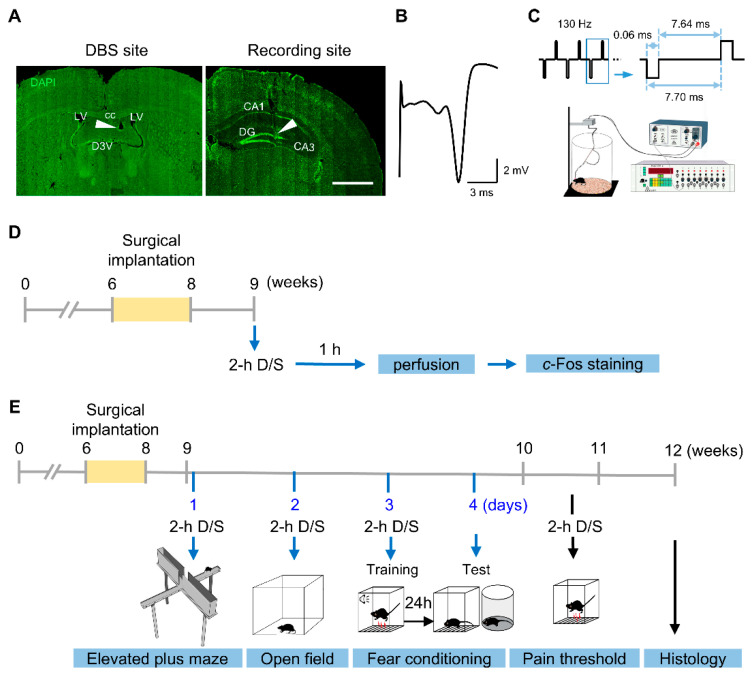
Forniceal DBS electrode implantation, parameter setting, and experimental design. (**A**) Photomicrographs illustrating DBS electrode placement (arrowhead) in the fimbria-fornix (*left*) and the recording electrode in the dentate gyrus (*right*). cc, corpus callosum; LV, lateral ventricle; D3V, dorsal third ventricle; DG, dentate gyrus. Scale bar, 500 μm. (**B**) Representative evoked potential trace of the fimbria-fornix pathway recorded in the dentate gyrus. (**C**) Schematic of the DBS setup (modified from Wang et al., 2023 [17]). (**D**) Schematic of the *c*-Fos experimental timeline. (**E**) Schematic timeline of mouse behavioral tests. 2-h D/S, 2 h of DBS or sham treatment.

DBS electrodes were implanted as previously described [7,8,16,17]. Briefly, mice were secured on a stereotaxic frame (David Kopf Instruments, Tujunga, CA, USA) under 1–2% isoflurane (Covetrus North America, Dublin, OH, USA) anesthesia and implanted with a bipolar stimulation electrode constructed with Teflon-coated tungsten wire (50 µm diameter, A-M system, Sequim, WA, USA). The electrodes were targeted unilaterally to the right fimbria-fornix under the guidance of evoked potentials recorded in the ipsilateral dentate gyrus [18] (Figure 1A,B). All electrode wires and the attached connector sockets were fixed on the skull with dental cement (C&B Metabond, Parkell Inc., Edgewood, NY, USA).

After at least 1 week of recovery, the mice were randomly assigned to the DBS or sham group. Animals in the DBS group received acute DBS (biphasic rectangular pulses, 130 Hz, 60 µs pulse duration, 50 µA pulse intensity) for 2 h under freely moving conditions in a Plexiglas chamber (Figure 1C). The 50 µA stimulus intensity was chosen based on the average of individually optimized DBS strength in the fornix of mice [7,8]. Sham-treated mice were implanted with electrodes but only attached to cables without DBS [7,16,17].

### 2.3. Immunohistochemical Staining

At the end of experiments, we followed previous studies [7,8,17] for mouse brain preparation, sectioning, and immunostaining of *c*-Fos (Figure 1D). The primary *c*-Fos monoclonal antibody MA5-15055 (1:250, ThermoFisher Scientific, Waltham, MA, USA) and the secondary antibody Alexa Fluor 568 goat anti-rabbit IgG (1:500, A11036, ThermoFisher Scientific, Waltham, MA, USA) were used in this study.

### 2.4. Imaging and Quantification

The digital images were collected by a laser scanning microscope LSM 880 (Zeiss, Oberkochen, Germany) and quantitatively analyzed using ImageJ software 1.47t (NIH). For all brain structures analyzed, *c*-Fos positive cells ipsilateral to DBS were counted and summed from four sections for each structure and then normalized to the sham group.

### 2.5. Behavioral Assays

An additional mouse cohort at 6–8 weeks of age was implanted with DBS electrodes. After recovery, the mice were randomly assigned into DBS or sham group. Behavioral tests were conducted immediately after 2 h of DBS or sham treatment (Figure 1C,E). Open field assay, elevated plus maze, fear conditioning, and pain threshold test were conducted following previous studies [7,8,17].

### 2.6. Histology

We followed previous methods for the histological evaluation of DBS cites [7,17]. A representative site of the electrode placement is shown in Figure 1A.

### 2.7. Statistical Analyses

Data were normalized to the sham group and analyzed using two-tailed student *t*-test if data distributed normally. The Mann–Whitney test was performed for non-normal distribution data. No data point was excluded from statistical analysis. *p* < 0.05 was the cut-off for statistical significance. Graphpad prism 9.5.0 and SigmaPlot 12.5 were used to create all the summarized plots and all the statistical tests in this study.

## 3. Results

**Figure 2:**
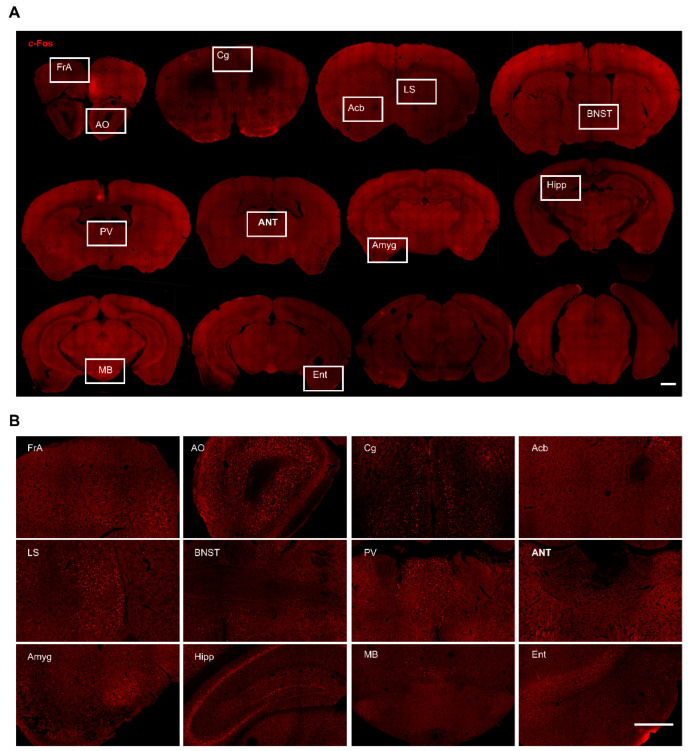
*c*-Fos expression in different brain regions in freely moving sham-treated mice. (**A**) Representative whole brain coronal sections (rostral to caudal) with *c*-Fos staining. Scale bar, 1 mm. (**B**) Magnified view of boxed areas in A. Scale bar, 500 μm. FrA, frontal association cortex; AO, anterior olfactory area; Cg, cingulate cortex; Acb, accumbent nucleus; LS, lateral septal nucleus; BNST, bed nucleus of stria terminalis; PV, paraventricular thalamic nucleus; ANT, anterior thalamic nucleus; Amyg, amygdala; Hipp, hippocampus; MB, mammillary body; Ent, entorhinal cortex.

**Figure 3:**
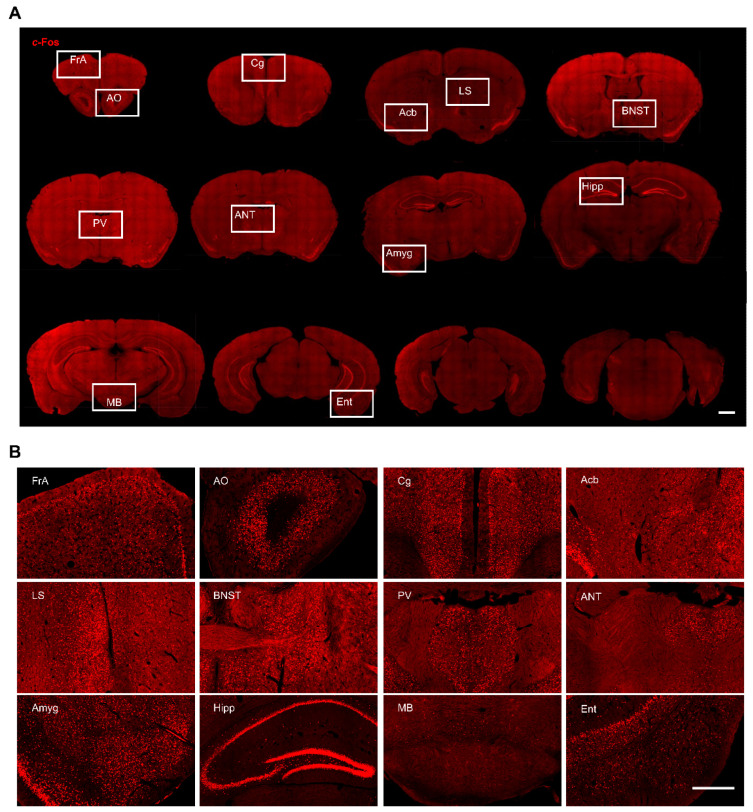
*c*-Fos expression in different brain regions in freely moving DBS-treated mice. (**A**) Representative whole brain coronal sections (rostral to caudal) with *c*-Fos staining. Scale bar, 1 mm. (**B**) Magnified view of boxed areas in (**A**). Scale bar, 500 μm.

To characterize the neural circuit forniceal DBS engages, we used *c*-Fos to identify anatomical structures in which neurons were activated following forniceal DBS. We mapped *c*-Fos expressions in whole brain coronal sections and compared those from DBS- and sham-treated mice. In general, we found scattered *c*-Fos positive neurons in the brain of sham mice (Figure 2) and robust *c*-Fos positive neurons in several brain sites bilaterally in DBS-treated mice (Figure 3). This is consistent with our previous study that unilateral forniceal DBS significantly increased the *c*-Fos expression in the dentate gyrus (DG) bilaterally without hemisphere difference [7]. The main purpose of this study was to explore the neural circuit involved in forniceal DBS. Therefore, we counted and compared *c*-Fos positive neurons in the brain between DBS- and sham-treated mice. We also evaluated the effects of acute forniceal DBS on animal behaviors.

### 3.1. c-Fos Expression in the Hippocampus

**Figure 4:**
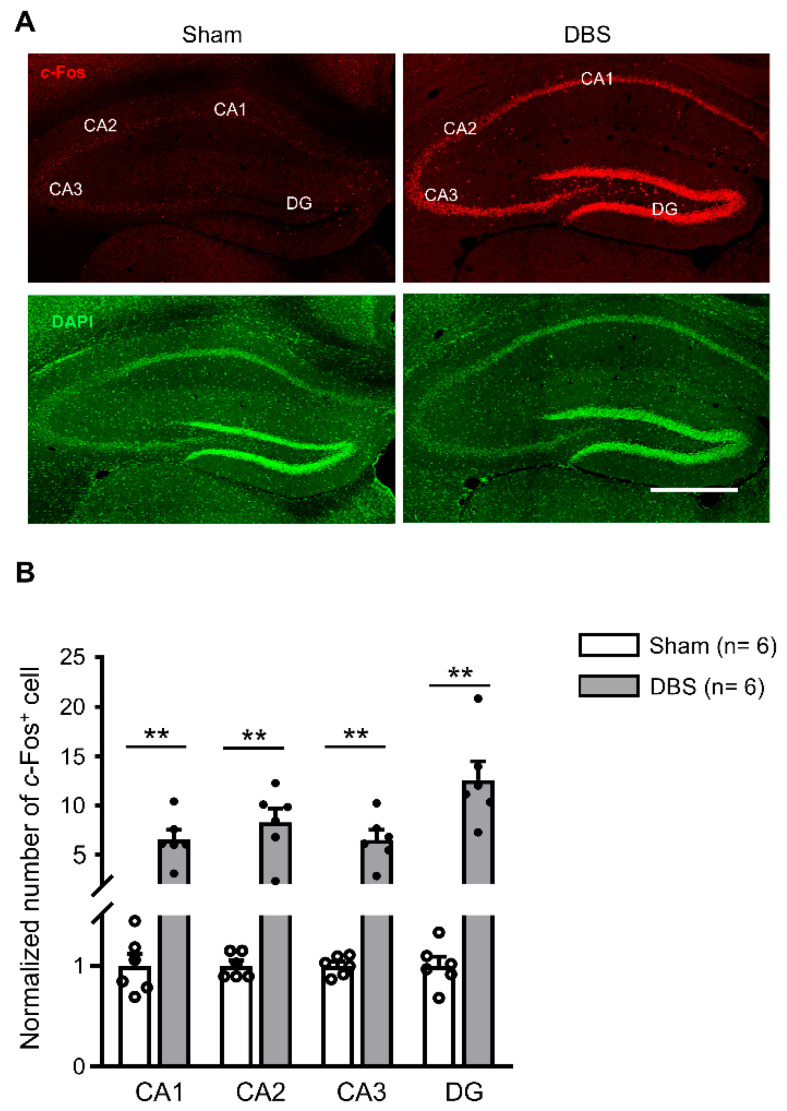
*c*-Fos expression in the hippocampus of sham- and DBS-treated mice. (**A**) Representative images showing *c*-Fos positive cells in the hippocampus of sham- and DBS-treated mice. Scale bar, 500 μm. DG, dentate gyrus. (**B**) Number of normalized *c*-Fos positive cells in the hippocampus of sham- and DBS-treated mice. Statistics: CA1 (sham 1.000 ± 0.115 vs. DBS 6.522 ± 0.976, Mann–Whitney test, *U* = 0.000, *p* = 0.002), CA2 (sham 1.000 ± 0.051 vs. DBS 8.277 ± 1.409, Mann–Whitney test, *U* = 0.000, *p* = 0.002), CA3 (sham 1.000 ± 0.039 vs. DBS 6.495 ± 0.999, Mann–Whitney test, *U* = 0.000, *p* = 0.002), and DG (sham 1.000 ± 0.088 vs. DBS 12.578 ± 1.877, Mann–Whitney test, *U* = 0.000, *p* = 0.002). ** *p* < 0.01. Data presented as mean ± SEM with individual values.

Since forniceal DBS enhances hippocampus-dependent learning and memory [5,6,7,8], we first evaluated *c*-Fos expression in the hippocampus. *c*-Fos positive neurons scattered in the hippocampus of sham-treated mice (Figure 4A). We found a significant increase in *c*-Fos expression in CA1, CA2, CA3, and DG in DBS-treated mice compared to sham-treated controls (Figure 4A,B).

### 3.2. c-Fos Expression in the Cortices

**Figure 5:**
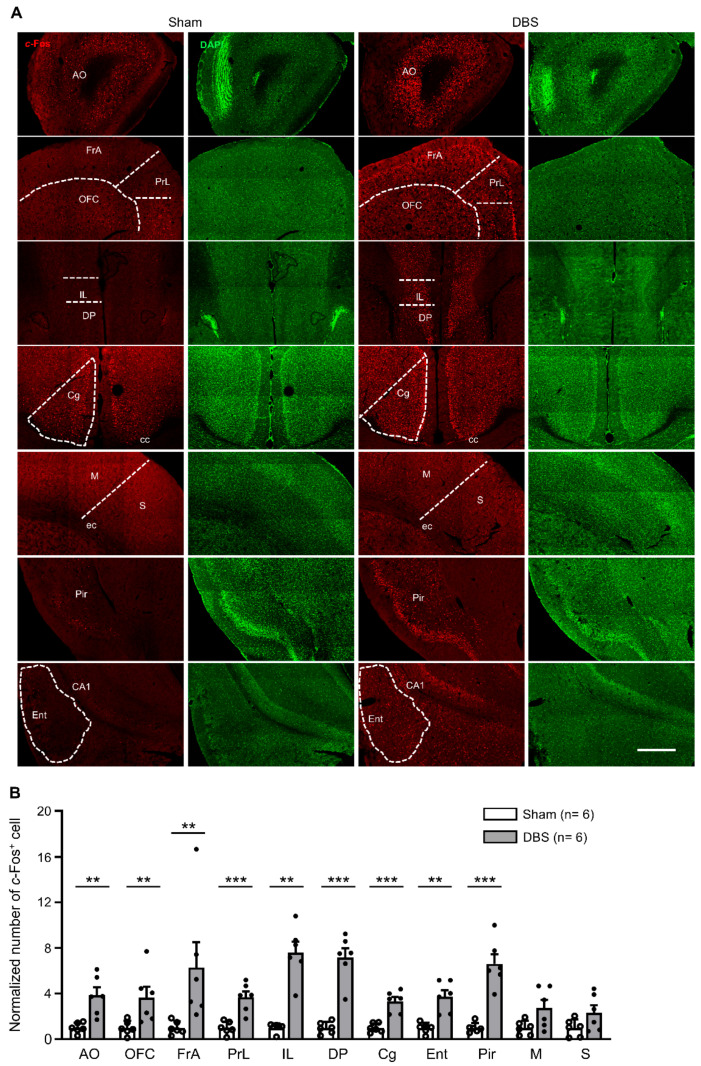
*c*-Fos expression in the cortices in sham- and DBS-treated mice. (**A**) Representative images of *c*-Fos positive cells in different cortices in sham- and DBS-treated mice. Scale bar, 500 μm. AO, anterior olfactory cortex; OFC, orbitofrontal cortex; FrA, frontal association cortex; PrL, prelimbic cortex; IL, infralimbic cortex; DP, dorsal peduncular cortex; Cg, cingulate cortex; Ent, entorhinal cortex; Pir, piriform cortex; M, motor cortex; S, somatosensory cortex; cc, corpus callosum; ec, external capsule. (**B**) Normalized number of *c*-Fos positive cells in different cortices in sham- and DBS-treated mice. Statistics: AO (sham 1.000 ± 0.184 vs. DBS 3.845 ± 0.698, two-tailed unpaired *t* test, *t*_(10)_ = −3.940, *p* = 0.003), OFC (sham 1.000 ± 0.615 vs. DBS 3.645 ± 0.947, Mann–Whitney test, *U* = 2.000, *p* = 0.009), FrA (sham 1.000 ± 0.231 vs. DBS 6.285 ± 2.241, Mann–Whitney test, *U* = 0.000, *p* = 0.002), PrL (sham 1.000 ± 0.227 vs. DBS 3.675 ± 0.512, two-tailed unpaired *t* test, *t*_(10)_ = −4.778, *p* = 0.0007), IL (sham 1.000 ± 0.163 vs. DBS 7.597 ± 0.949, Mann–Whitney test, *U* = 0.000, *p* = 0.002), DP (sham 1.000 ± 0.210 vs. DBS 7.166 ± 0.823, two-tailed unpaired *t* test, *t*_(10)_ = −7.264, *p* = 0.00003), Cg (sham 1.000 ± 0.161 vs. DBS 3.313 ± 0.381, two-tailed unpaired *t* test, *t*_(10)_ = −5.591, *p* = 0.0002), Ent (sham 1.000 ± 0.186 vs. DBS 3.737 ± 0.552, Mann–Whitney test, *U* = 0.000, *p* = 0.002), Pir (sham 1.000 ± 0.175 vs. DBS 6.612 ± 0.842, two-tailed unpaired *t* test, *t*_(10)_ = −6.524, *p* = 0.00007), M (sham 1.000 ± 0.254 vs. DBS 2.744 ± 0.715, Mann–Whitney test, *U* = 7.000, *p* = 0.093) and S (sham 1.000 ± 0.304 vs. DBS 2.333 ± 0.649, Mann–Whitney test, *U* = 10.000, *p* = 0.240). ** *p* < 0.01, *** *p* < 0.001. Data presented as mean ± SEM with individual values.

We counted *c*-Fos positive neurons in the anterior olfactory cortex (AO), orbitofrontal cortex (OFC), frontal association cortex (FrA), prelimbic cortex (PrL), infralimbic cortex (IL), dorsal peduncular cortex (DP), cingulate cortex (Cg), entorhinal cortex (Ent), piriform cortex (Pir), motor cortex (M), and somatosensory cortex (S) (Figure 5A). Compared to sham mice, DBS significantly increased *c*-Fos expression in AO, OFC, FrA, PrL, IL, DP, Cg, Ent, and Pir, respectively. However, we found no difference in *c*-Fos positive cell numbers in M and S between DBS- and sham-treated groups (Figure 5B). 

### 3.3. c-Fos Expression in the Subcortical Regions

**Figure 6:**
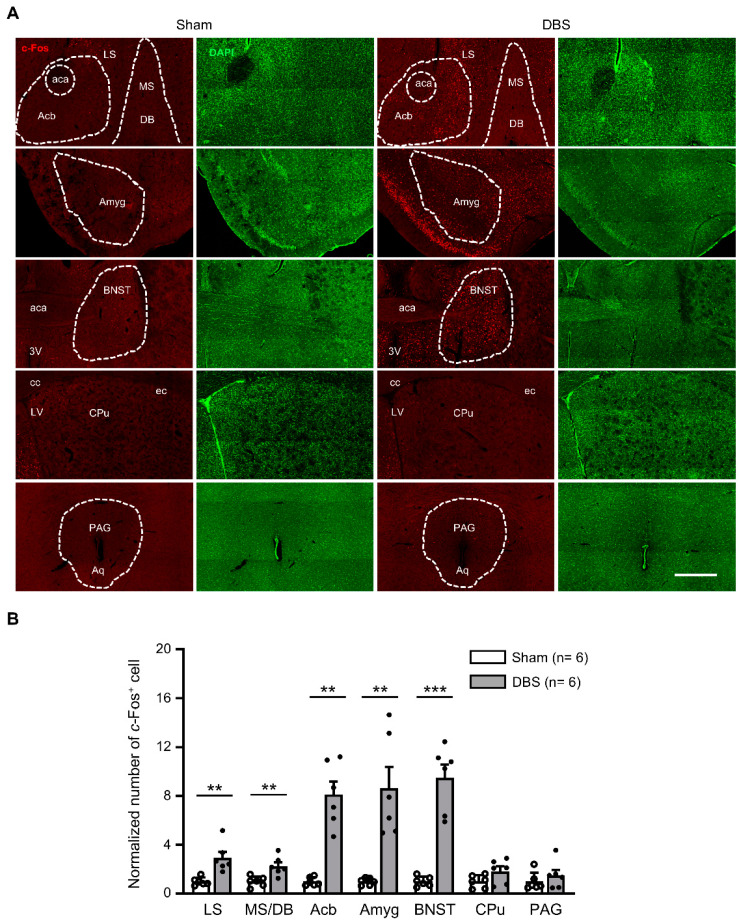
*c*-Fos expression in the subcortical regions in sham- and DBS-treated mice. (**A**) Representative images of *c*-Fos positive cells in the subcortical regions in the sham- and DBS-treated mice. Scale bar, 500 μm. LS, lateral septal nucleus; MS/DB, medial septal nucleus/nucleus of the diagonal band; Acb, accumbent nucleus; BNST, bed nucleus of stria terminalis; Cpu, caudate putamen; Amyg, amygdala; PAG, periaqueductal gray; aca, anterior commissure, anterior part; cc, corpus callosum; ec, external capsule; 3V, 3rd ventricle; Aq, aqueduct. (**B**) Normalized number of *c*-Fos positive cells in the subcortical regions in sham- and DBS-treated mice. Statistics: LS (sham 1.000 ± 0.135 vs. DBS 2.950 ± 0.491, Mann–Whitney test, *U* = 0.000, *p* = 0.002), MS/DB (sham 1.000 ± 0.171 vs. DBS 2.264 ± 0.322, two-tailed unpaired *t* test, *t*_(10)_ = −3.468, *p* = 0.006), Acb (sham 1.000 ± 0.140 vs. DBS 8.135 ± 1.074, Mann–Whitney test, *U* = 0.000, *p* = 0.002), Amyg (sham 1.000 ± 0.118 vs. DBS 8.670 ± 1.719, Mann–Whitney test, *U* = 0.000, *p* = 0.002), BNST (sham 1.000 ± 0.166 vs. DBS 9.484 ± 1.103, two-tailed unpaired *t* test, *t*_(10)_ = −7.608, *p* = 0.0002), CPu (sham 1.000 ± 0.216 vs. DBS 1.843 ± 0.393, two-tailed unpaired *t* test, *t*_(10)_ = −1.883, *p* = 0.089) and PAG (sham 1.000 ± 0.301 vs. DBS 1.503 ± 0.458, Mann–Whitney test, *U* = 13.500, *p* = 0.485). ** *p* < 0.01, *** *p* < 0.001. Data presented as mean ± SEM with individual values.

*c*-Fos positive neurons were counted in the lateral septal nucleus (LS), medial septum-diagonal band of Broca (MS/DB), accumbent nucleus (Acb), amygdala (Amyg), bed nucleus of stria terminalis (BNST), caudate putamen (CPu), and periaqueductal gray (PAG) (Figure 6A). Compared to sham mice, DBS significantly enhanced *c*-Fos expression in the LS, MS/DB, Acb, Amyg, and BNST, respectively. There was no change in *c*-Fos positive cell count in the CPu and PAG following DBS (Figure 6B).

### 3.4. c-Fos Expression in the Thalamic Nuclei

**Figure 7:**
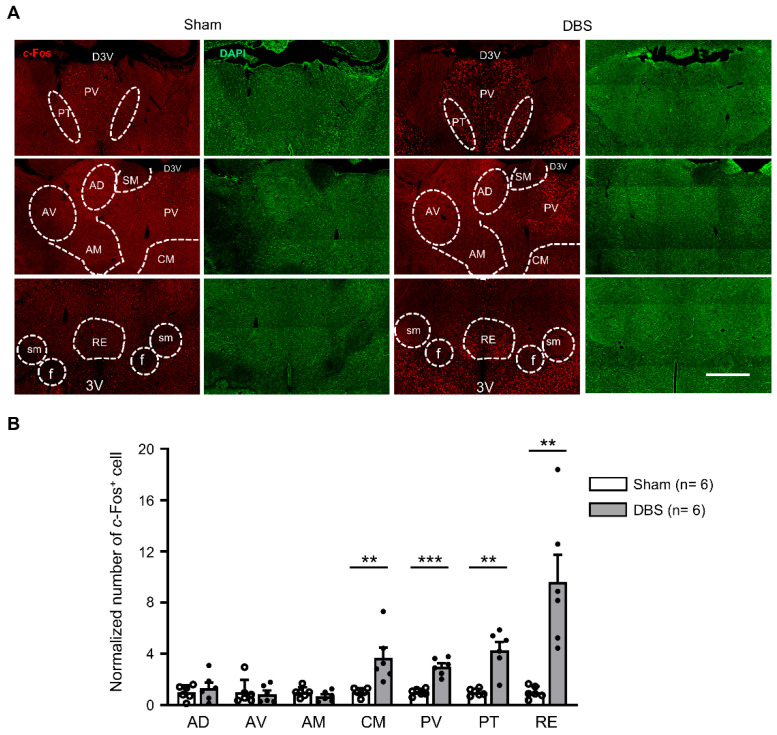
*c*-Fos expression in the thalamic nuclei in sham- and DBS-treated mice. (**A**) Representative images of *c*-Fos positive cells in the thalamic nuclei in the sham- and DBS-treated mice. Scale bar, 500 μm. AD, anterodorsal thalamic nucleus; AV, anteroventral thalamic nucleus; AM, anteromedial thalamic nucleus; CM, central medial thalamic nucleus; PV, paraventricular thalamic nucleus; PT, paratenial thalamic nucleus; RE, reuniens thalamic nucleus; D3V, dorsal 3rd ventricle; 3V, 3rd ventricle; f, fornix; sm, stria medullaris. (**B**) Normalized number of *c*-Fos positive cells in the thalamic nuclei in sham- and DBS-treated mice. Statistics: AD (sham 1.000 ± 0.225 vs. DBS 1.333 ± 0.426, two-tailed unpaired *t* test, *t*_(10)_ = −0.692, *p* = 0.505), AV (sham 1.000 ± 0.399 vs. DBS 0.863 ± 0.272, Mann–Whitney test, *U* = 15.500, *p* = 0.699), AM (sham 1.000 ± 0.394 vs. DBS 0.690 ± 0.398, two-tailed unpaired *t* test, *t*_(10)_ = 1.355, *p* = 0.205), CM (sham 1.000 ± 0.130 vs. DBS 3.667 ± 0.807, Mann–Whitney test, *U* = 0.000, *p* = 0.002), PV (sham 1.000 ± 0.111 vs. DBS 2.989 ± 0.273, two-tailed unpaired *t* test, *t*_(10)_ = −6.747, *p* = 0.00005), PT (sham 1.000 ± 0.107 vs. DBS 4.280 ± 0.636, Mann–Whitney test, *U* = 0.000, *p* = 0.002), RE (sham 1.000 ± 0.191 vs. DBS 9.602 ± 2.116, Mann–Whitney test, *U* = 0.000, *p* = 0.002). ** *p* < 0.01, *** *p* < 0.001. Data presented as mean ± SEM with individual values.

We counted *c*-Fos positive neurons in seven thalamic nuclei (Figure 7A). Compared to sham mice, DBS significantly increased *c*-Fos expression in the central medial thalamic nucleus (CM), paraventricular thalamic nucleus (PV), paratenial thalmic nucleus (PT), and reuniens thalamic nucleus (RE), respectively. In contrast, there was no change in the number of *c*-Fos positive cells found in the anterodorsal thalamic nucleus (AD), anteroventral thalamic nucleus (AV), and anteromedial thalamic nucleus (AM) (Figure 7B).

### 3.5. c-Fos Expression in the Hypothalamic Nuclei

**Figure 8:**
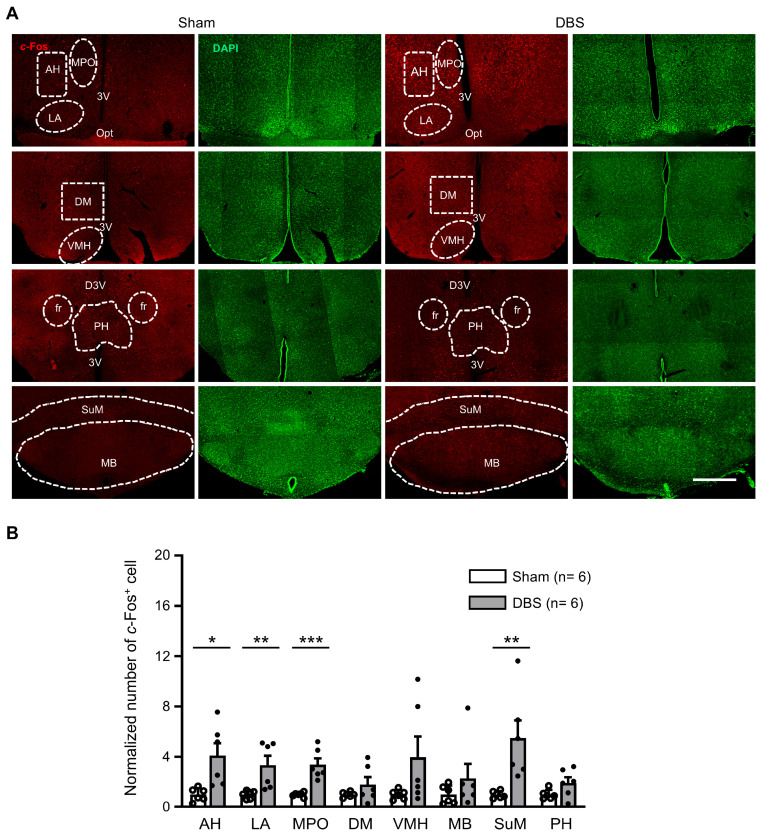
*c*-Fos expression in the hypothalamic nuclei in sham- and DBS-treated mice. (**A**) Representative images of *c*-Fos positive cells in the hypothalamus nuclei in the sham- and DBS-treated mice. Scale bar, 500 μm. AH, anterior hypothalamic area, anterior part; LA, lateral anterior hypothalamic nucleus; MPO, medial preoptic nucleus; DM, dorsomedial hypothalamic nucleus; VMH, ventromedial hypothalamic nucleus; PH, posterior hypothalamic nucleus; SuM, supramammillary nucleus; MB, mammillary body; Opt, optic tract; 3V, 3rd ventricle; D3V, dorsal 3rd ventricle. (**B**) Normalized number of *c*-Fos positive cells in the hypothalamic nuclei in sham- and DBS-treated mice. Statistics: AH (sham 1.000 ± 0.214 vs. DBS 4.106 ± 0.984, two-tailed unpaired *t* test, *t*_(10)_ = −3.085, *p* = 0.0115), LA (sham 1.000 ± 0.132 vs. DBS 3.337 ± 0.747, Mann–Whitney test, *U* = 0.000, *p* = 0.002), MPO (sham 1.000 ± 0.073 vs. DBS 3.386 ± 0.492, two-tailed unpaired *t* test, *t*_(10)_ = −4.795, *p* = 0.0007), DM (sham 1.000 ± 0.084 vs. DBS 1.804 ± 0.593, two-tailed unpaired *t* test, *t*_(10)_ = −1.343, *p* = 0.209), VMH (sham 1.000 ± 0.153 vs. DBS 3.966 ± 1.656, two-tailed unpaired *t* test, *t*_(10)_ = −1.783, *p* = 0.105), MB (sham 1.000 ± 0.301 vs. DBS 2.301 ± 1.140, Mann–Whitney test, *U* = 12.000, *p* = 0.394), SuM (sham 1.000 ± 0.117 vs. DBS 5.489 ± 1.406, Mann–Whitney test, *U* = 0.000, *p* = 0.002), and PH (sham 1.000 ± 0.154 vs. DBS 1.930 ± 0.453, two-tailed unpaired *t* test, *t*_(10)_ = −1.942, *p* = 0.081). * *p* < 0.05, ** *p* < 0.01, *** *p* < 0.001. Data presented as mean ± SEM with individual values.

We counted *c*-Fos positive neurons in eight hypothalamic nuclei (Figure 8A). Compared to sham mice, DBS significantly increased *c*-Fos expression in the anterior hypothalamic nucleus (AH), lateral anterior hypothalamic nucleus (LA), medial preoptic nucleus (MPO), and supramammillary nucleus (SuM), respectively. There was no difference in the number of *c*-Fos positive cell count in the dorsomedial hypothalamic nucleus (DM), ventromedial hypothalamic nucleus (VMH), mammillary body (MB), and posterior hypothalamic nucleus (PH) (Figure 8B).

### 3.6. Behavioral Effect of Acute Forniceal DBS

**Figure 9:**
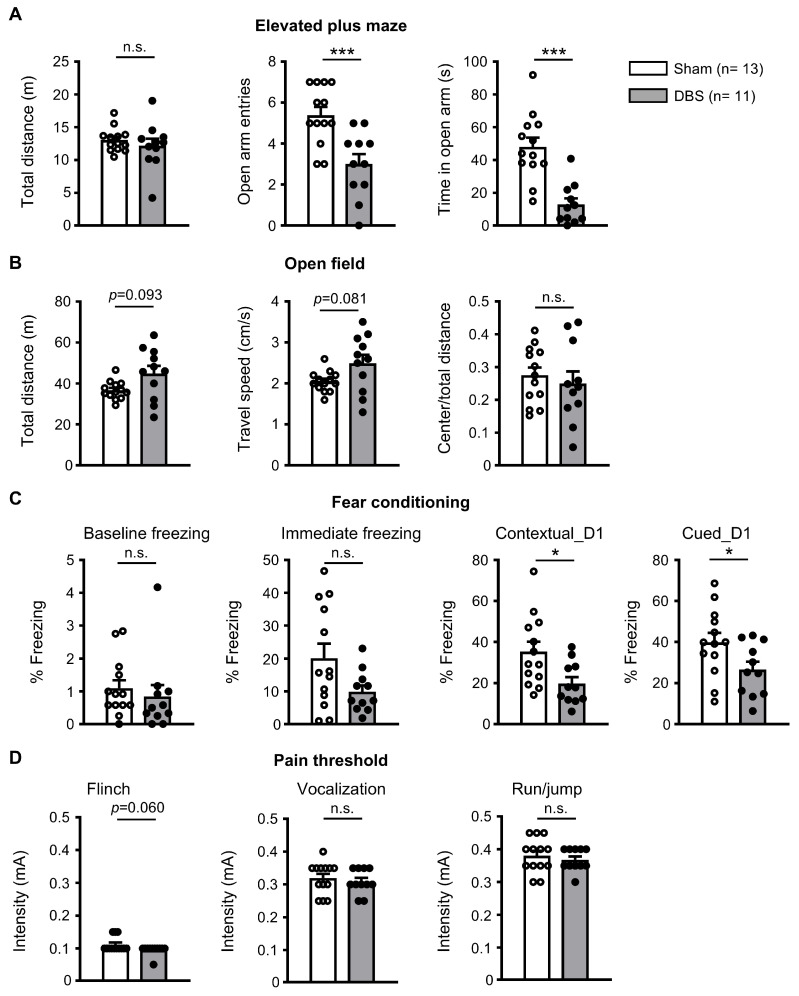
Behavioral effects of acute forniceal DBS in mice. (**A**) Anxiety was tested in the elevated plus maze. Statistics: Travel distance (sham 13.045 ± 0.506 m vs. DBS 12.177 ± 1.081 m, Mann–Whitney test, *U* = 61.000, *p* = 0.562), the number of open arm entries (sham 5.385 ± 0.401 vs. DBS 3.000 ± 0.486, two-tailed unpaired *t* test, *t*_(22)_ = 3.820, *p* = 0.0009), and time in open arm (sham 48.062 ± 5.605 s vs. DBS 12.864 ± 3.701 s, two-tailed unpaired *t* test, *t*_(22)_ = 5.034, *p* = 0.00002). (**B**) Motor function was tested in open-field assay. Statistics: Travel distance (sham 36.721 ± 1.226 m vs. DBS 44.877 ± 3.789 m, Mann–Whitney test, *U* = 42.000, *p* = 0.093), travel speed (sham 2.046 ± 0.0704 cm/s vs. DBS 2.491 ± 0.211 cm/s, Mann–Whitney test, *U* = 41.000, *p* = 0.081), and the ratio of center/total distance (sham 0.275 ± 0.024 vs. DBS 0.250 ± 0.037, two-tailed unpaired *t* test, *t*_(22)_ = 0.585, *p* = 0.564). (**C**) Contextual and cued memory were assessed in fear conditioning. Statistics: Baseline freezing before the first foot shock (sham 1.096 ± 0.245% vs. DBS 0.841 ± 0.352%, Mann–Whitney test, *U* = 50.000, *p* = 0.221), immediate freezing after the second foot shock (sham 20.154 ± 4.336% vs. DBS 9.909 ± 1.900%, Mann–Whitney test, *U* = 46.000, *p* = 0.147), contextual freezing (sham 35.292 ± 4.816% vs. DBS 19.836 ± 3.098%, two-tailed unpaired *t* test, *t*_(22)_ = 2.588, *p* = 0.017, cued freezing (sham 39.813 ± 4.605% vs. DBS 26.642 ± 3.873%, two-tailed unpaired *t* test, *t*_(22)_ = 2.141, *p* = 0.044). (**D**) Pain threshold was evaluated by flinch, vocalization, or run/jumping test. Statistics: current intensity for flinch: sham 0.112 ± 0.006 mA vs. DBS 0.096 ± 0.005 mA, Mann–Whitney test, *U* = 50.000, *p* = 0.060; vocalization: sham 0.319 ± 0.013 mA vs. DBS 0.309 ± 0.011 mA, Mann–Whitney test, *U* = 61.500, *p* = 0.558; run/jump: sham 0.381 ± 0.015 mA vs. DBS 0.368 ± 0.010 mA, two-tailed unpaired *t* test, *t*_(22)_ = 0.687, *p* = 0.5). * *p* < 0.05, *** *p* < 0.001, n.s. not significant. Data presented as mean ± SEM with individual values.

Chronic forniceal DBS has been reported to improve learning and memory in mouse models of Rett syndrome and CDKL5 deficiency disorders [7,8]. We wondered whether acute forniceal DBS would have any behavioral effects. Therefore, we performed multiple behavioral assays in mice immediately after 2 h forniceal DBS or sham treatment (Figure 1E and Figure 9). We first evaluated anxiety behavior via an elevated plus maze test. As shown in Figure 9A, although DBS- and sham-treated mice showed similar travel distance, the number of open arm entries and time in open arm were significantly less in DBS-treated mice, suggesting that acute forniceal DBS increased anxiety. Then, we tested the locomotion using an open field assay (Figure 9B). There was no difference between DBS- and sham-treated mice in travel distance, travel speed, and the ratio of center/total distance, indicating that acute DBS did not change locomotion behavior. Finally, we evaluated the effect of acute DBS on contextual and cued fear memory (Figure 9C). DBS- and sham-treated mice showed similar levels of baseline freezing before the first foot shock and immediate freezing after the second foot shock. However, DBS-treated mice froze less in both the conditioning context and the cued novel environment than sham-treated mice when tested 24 h after fear conditioning training. There was no difference in pain threshold (Figure 9D) between DBS- and sham-treated mice. These fear memory results suggest that acute forniceal DBS might suppress learning and memory.

## 4. Discussion

The fornix is a bundle of nerve fibers that connects the hippocampus with many brain structures. Forniceal DBS may act through orthodromic and antidromic axons to modulate neuronal activity in the central nervous system. To determine which neural circuits are related to forniceal DBS, we characterized the brain regions showing *c*-Fos expression in mice after exposing them to acute forniceal DBS and compared that with sham-treated mice. We found that multiple brain regions showed a significantly increased number of cells with *c*-Fos expression after acute forniceal DBS. Thus, our data supports the hypothesis that forniceal DBS introduced a new pattern of neural network activity in the brain. Below we summarize the major brain structures and neural circuit pathways activated by DBS, each followed by a brief discussion. We intentionally focus on the neural circuits involving the hippocampus because forniceal DBS improves hippocampal learning and memory [5,6,7,8,17].

### 4.1. Hippocampal Circuit

The hippocampal formation is crucial to memory function. It has a unique anatomical structure and can be divided into several subregions, including CA1, CA2, CA3, and DG, based on morphological and physiological properties. There is a trisynaptic circuit in the hippocampal formation: the granule cells in DG receive cortical excitatory input through the perforant path and send their mossy fiber axons to form excitatory synapses on the dendrites of CA3 pyramidal neurons. Then, CA3 pyramidal neurons project onto CA1 pyramidal neurons via the Schaffer collateral pathway. Intensive studies have established that the hippocampal subregions serve cooperative and distinctive functions in memory information processing. The CA1 is primarily associated with retrieving and consolidating episodic memories; CA3 is involved in pattern completion and spatial pattern separation; and the DG plays a key role in initial encoding and distinguishing between similar memories (pattern separation) by filtering out irrelevant information [19,20,21,22,23,24,25,26]. In addition, CA2 sends excitatory projections to all other hippocampal regions and receives input from the entorhinal cortex and all other hippocampal regions except for CA1. CA2 has a fundamental role in hippocampal information processing [27].

The fornix is responsible for the major efferent and afferent projections of the hippocampus. It has been reported that fornix stimulation produces robust, short-latency hippocampal evoked potentials in mice [7,8,16], rats [28], and ovine [29]. As expected, robust *c*-Fos expression was found in all hippocampal subfields (CA1, CA2, CA3, and DG) in DBS-treated mice compared to sham-treated controls. Together, these results suggest that acute forniceal DBS excites the hippocampal circuitry, indicating that the hippocampus plays a critical role in forniceal DBS-induced memory benefits.

### 4.2. Cortico-Hippocampal Circuit

Although the hippocampus plays an important role in learning and memory, it does not act in isolation. Hippocampal neurons communicate extensively with the cortical regions to process memory information. The entorhinal cortex (Ent) is commonly perceived as a major input and output structure of the hippocampal formation, forming the nodal point of cortico-hippocampal circuits [30,31]. The prefrontal cortex (PFC) and olfactory cortex have provided inputs to the hippocampus relayed at Ent [32,33]. The PFC is anatomically divided into different regions (medial, lateral, and orbital), two of which are important for learning and memory: the medial prefrontal cortex (mPFC, usually comprising the anterior cingulate cortex (Cg), prelimbic cortex (PL), infralimbic cortex (IL), and dorsal peduncular cortex (DP)) and the orbitofrontal cortex [34,35,36].

The frontal association cortex (FrA) is composed of the PFC and all motor-related areas except for the primary motor cortex [37]. FrA receives wide afferent projections from the whole brain, including other parts of PFC, amygdala, thalamus, and hippocampus [38,39]. FrA is implicated in various higher brain functions, such as associative learning [40].

The olfactory cortex includes the anterior olfactory nucleus (AO) and piriform cortex (Pir). The olfactory and hippocampal systems are closely and reciprocally interconnected [41,42]. Olfaction, the sense of smell, is closely associated with learning and memory.

In this study, we found that acute forniceal DBS significantly increased *c*-Fos expression in Ent, Cg, PL, IL, DP, OFC, FrA, AO, and Pir but not in the motor or somatosensory cortex. These data suggest that acute forniceal DBS activates Ent, PFC, FrA, and the olfactory cortex, which are the core components of the cortico-hippocampal circuit.

### 4.3. Septo-Hippocampal Circuit

The fornix is divided into the pre-commissural and post-commissural fornix around the anterior commissure [2]. The pre-commissural fornix is the main neural pathway between the hippocampus and septal area [43]. The septal area is anatomically and functionally divided into two major regions: lateral septal nuclei (LS) and medial septum/diagonal band of Broca nuclei (MS/DB) [44,45]. The LS is a major relay that connects the hippocampus to multiple subcortical regions, such as the hypothalamic area, medial preoptic nucleus (MPO), supramammillary nucleus (SuM), MS, accumbent nucleus (Acb), and PAG [46,47]. Its central position makes the LS a hub for integrating a variety of effects, such as reward, feeding, anxiety, fear, sociability, and memory [48]. In parallel, the MS/DB is an important modulator of hippocampal function. The hippocampus not only receives both cholinergic and GABAergic input from MS/DB [49], but also projects back to the MS/DB [50,51,52]. Therefore, they form a reciprocal long-range GABAergic septo-hippocampal circuit [53], which has been proven to play a role in learning and memory [44]. It has been reported that brief electrical stimuli to the fornix can evoke neural activity in rat septum [54,55]. In this study, we found that acute forniceal DBS significantly enhances *c*-Fos expression in LS and MS/DB. These data suggest that the septo-hippocampal circuit is activated after acute forniceal DBS and may contribute to memory benefits.

### 4.4. Hippocampal-Hypothalamic Circuit

The hypothalamus is a small but important area at the bottom of the brain, which consists of several small nuclei and serves as a link between the nervous system and the endocrine system. It controls several vital functions, including homeostasis and feeding. As an important part of the limbic system, the hypothalamus reciprocally interconnects with hippocampus [56,57] and might act as an interface for various cognitive functions [58]. It has been reported that the anterior hypothalamic nucleus (AH) mediates the contextual memory of predator threats [59] and emotional behaviors [60,61]. The lateral hypothalamic nucleus (LA) plays an important role in food-reward learning and memory and feeding behavior control [58]. The medial preoptic nucleus (MPO) is involved in the emotional processing of olfactory stimuli in rodents [62,63] and undergoes plastic changes [64] related to forming long-term memories. The supramammillary nucleus (SuM) is a thin layer of cells in the brain that lies above the mammillary bodies in the caudal hypothalamus and directly projects to the hippocampus [65]. Lesion to the fornix significantly decreases *c*-Fos expression in SuM [66,67]. The SuM exerts a pronounced influence on hippocampal learning and memory [68,69,70,71]. In this study, we found that forniceal DBS significantly increased *c*-Fos expression in AH, LA, MPO, and SuM compared to sham-treated mice, but not in the dorsomedial hypothalamic nucleus (DM), ventromedial hypothalamic nucleus (VMH), or posterior hypothalamic nucleus (PH). This panel of discoveries indicates that acute forniceal DBS activates AH, LA, MPO, and SuM.

As a part of the hypothalamus, the mammillary body (MB) receives inputs from the hippocampus via the post-commissural fornix and projects onto the anterior thalamic nuclei through the mammillothalamic tract [72,73,74,75]. Unexpectedly, we found very few *c*-Fos positive neurons in MB and there was no difference between the DBS- and sham-treated mice. Vann et al. revealed that *c*-Fos expression in MB was too low to be counted following a spatial memory task in their independent studies [66]. Notably, it has been reported that the contribution of MB to memory is more dependent on afferents from the ventral tegmental nucleus of Gudden than from the hippocampal formation [76]. These findings are consistent with our results. In addition, in this study, the DBS target was the pre-commissural fornix. So, considering our bipolar stimulation and low stimulus intensity of the DBS setting, the post-commissural fornix might not have been stimulated.

### 4.5. Hippocampal–Thalamic Circuit

The thalamus is a group of nuclei located in the center of the brain and is mainly responsible for relaying different sensory signals to the cerebral cortex and regulating consciousness, sleep, and alertness [77]. Among the thalamic nuclei, the anterior thalamic nuclei are a vital node within hippocampal–diencephalic–cingulate circuits that support spatial learning and memory [78]. The anterior thalamic nuclei, composed of the anteromedial (AM), anteroventral (AV), and anterodorsal nuclei (AD), connect to the hippocampus via the mammillothalamic tract, which joins the mammillary bodies and fornix [79]. Given the extensive direct and indirect interconnection with the fornix and hippocampus, we expected an increase in neuronal activity in the anterior thalamic nuclei after forniceal DBS. Surprisingly, there was no difference in *c*-Fos expression in AM, AV, and AD between DBS- and sham-treated mice, suggesting that the anterior thalamic nuclei might not be involved in the neural circuits activated by acute forniceal DBS. This result indirectly supports the proposition that acute forniceal DBS did not activate the post-commissural fornix or mammillary body in this study.

The midline thalamic nuclei are principally composed of the paraventricular (PV), paratenial (PT), and reuniens thalamic nucleus (RE). PV and PT mainly project to the subcortical limbic structures, particularly the amygdala and nucleus accumbens, and hence, are critically involved in affective behaviors such as stress/anxiety, feeding behavior, and drug-seeking activities [80]. As the largest of the midline nuclei, the RE provides the strongest thalamic input to the hippocampal formation [81]. The central medial thalamic nucleus (CM) is a poorly understood component of the thalamus. CM projections to the limbic and sensorimotor structures of the rostral forebrain suggest that CM may participate in integrating affective, cognitive, and sensorimotor functions [82]. We found that forniceal DBS significantly increased *c*-Fos expression in the PV, PT, RE, and CM, indicating that PV, PT, RE, and CM were activated by acute forniceal DBS.

### 4.6. Hippocampal-Amygdala Circuit

The amygdala plays a key role in emotional processing. Anatomical investigations have revealed that the amygdala and hippocampus are reciprocally connected [83,84]. Studies in animals indicate that the enhancing effect of emotional arousal on memory consolidation depends on the influence of amygdala on the hippocampus [85,86,87].

The bed nucleus of stria terminalis (BNST), also referred as the extended amygdala, is interconnected with essential emotional processing regions, including the hippocampus and amygdala [88] and involved in the modulation of fear memory [89,90,91].

We found that forniceal DBS significantly increased *c*-Fos expression in the amygdala and BNST. These results indicate that the hippocampal–amygdala circuit can be activated by acute forniceal DBS. Relatedly, forniceal DBS enhances amygdala-dependent cued fear memory in *Cdkl5* mutant mice [8].

### 4.7. Hippocampal–Accumbens Circuit

The nucleus accumbens (Acb), a component of basal ganglia that is critical to reward and motivation, receives glutamatergic projections from the amygdala [92] and hippocampus [93]. Acb is considered to be an interface between cognition, emotion, and action [94]. It has been reported that acute forniceal DBS enhances neural activity and dopamine release in the Acb [95,96]. We found that forniceal DBS significantly increased the *c*-Fos expression in Acb, indicating that the hippocampal–accumbens pathway can be activated by acute forniceal DBS.

### 4.8. Hippocampal-Striatal Circuit

The caudate putamen (CPu), a central component of the basal ganglia, has been thought to be predominantly involved in motor functions, but is now also known to play important roles in cognition [97]. In this study, we observed no significant change in *c*-Fos expression, indicating that the CPu was not activated by acute forniceal DBS. This finding is consistent with behavioral tests in this (Figure 9B) and previous studies [7], in which forniceal DBS did not change the locomotion level and motor function in mice.

### 4.9. Pain Descending Modulation Circuit

The periaqueductal gray (PAG) is a central brain region that plays an important role in descending pain modulation [98]. PAG has also been implicated in aversive learning. It receives information from aversive-signaling sensory systems and sends ascending projections to the thalamus and other forebrain structures that could control learning and memory [99,100]. In this study, we observed no significant difference in *c*-Fos expression in the PAG between DBS- and sham-treated mice. This indicates that PAG might not be involved in the neural circuits affected by forniceal DBS, which is consistent with behaviors tested in this (Figure 9D) and previous studies showing that forniceal DBS did not change the pain threshold in mice [7,8,17].

### 4.10. The Effect of Acute Forniceal DBS on Emotion and Memory

The limbic system is a group of interconnected brain structures that are associated with sensation, emotion, and memory. Its key components include the amygdala, hippocampus, thalamus, hypothalamus, septal area, and cingulate gyrus. It has been reported that individuals with anxiety disorders have increased neural activity in these brain areas [101,102,103]. As a result, anxiety disorders exert negative effects on memory [104]. In this study, we found that acute forniceal DBS robustly increased *c*-Fos expression in most components of the mouse limbic system, and DBS-treated mice showed increased anxiety levels and decreased fear memory, which is consistent with the negative correlation between anxiety and memory [104]. It is possible that acute forniceal DBS induces hyperactivity in the limbic system, resulting in increased anxiety and memory loss. Therefore, the forniceal DBS parameters should be optimized in different animal models to obtain therapeutic effects by properly toning the neural activity of the limbic system. Notably, chronic forniceal DBS improves hippocampal learning and memory [7,8,17]. It is possible that long-term DBS may reshape the neuronal morphological and/or functional properties of the larger scale neural network. The current study helps understand how acute/brief DBS impacts the neural network activity towards the chronic modifications of the overall neural circuitry and behavior.

**Figure 10:**
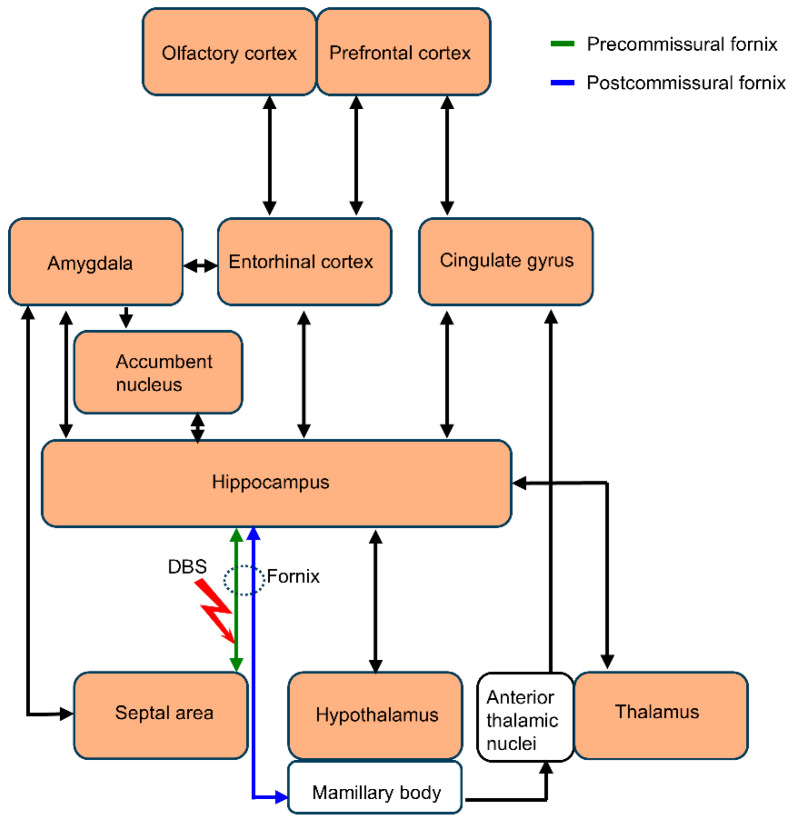
Schematic representation of the main structures and pathways being activated (color-filled rectangles) by acute forniceal DBS in mouse brain.

In summary, acute forniceal DBS activates multiple limbic structures associated with emotion and memory. The interaction of several neural circuits (Figure 10) may contribute to the effect of forniceal DBS. The neural circuits revealed here in wild-type mice help elucidate the neural network effect and pave the way for further research on the mechanism by which forniceal DBS induces benefits in animal models of cognitive impairments.

## Data Availability

The original contributions presented in this study are included in the article. Further inquiries can be directed to the corresponding author.
